# Supplementary material for: Demonstration of the effect of stirring on nucleation from experiments on the International Space Station using the ISS-EML facility
Source: NPJ Microgravity. 2021 Aug 6;7:31. doi: 10.1038/s41526-021-00161-9 (PMC8346615; doi:10.1038/s41526-021-00161-9)
Supplement: Supplementary file 1 — Supplementary Information [file 41526_2021_161_MOESM1_ESM.pdf]

SUPPLEMENTAL

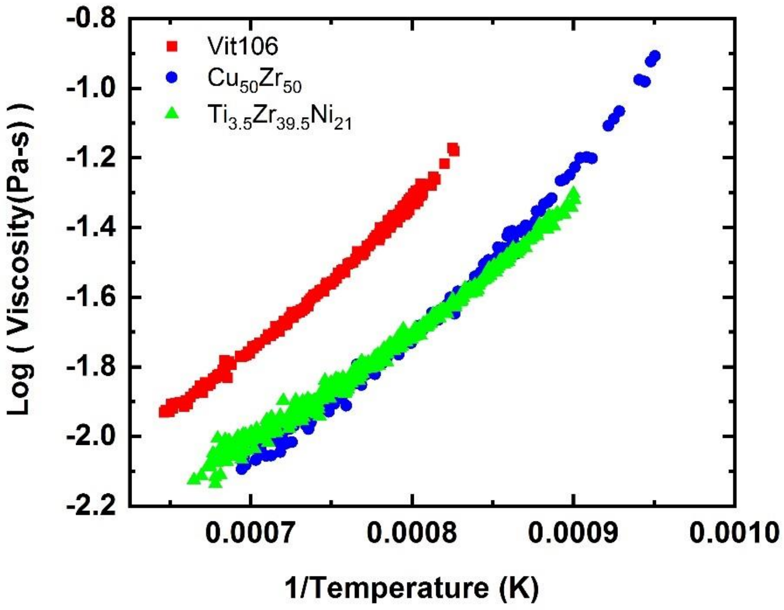

Supplementary Figure 1. The viscosities of three different liquids as a function of temperature measured by the ESL technique.

The fluid flows and shear rates in the liquids were estimated following procedures described in refs. [37,38]. Supplementary Figure 2 shows the results for fluid flows for one such calculation for the $Ti_{39.5}Zr_{39.5}Ni_{21}$ liquid at the nucleation temperature of 990 K, when it was cooled in vacuum with 5.7 V positioner and heater off condition.

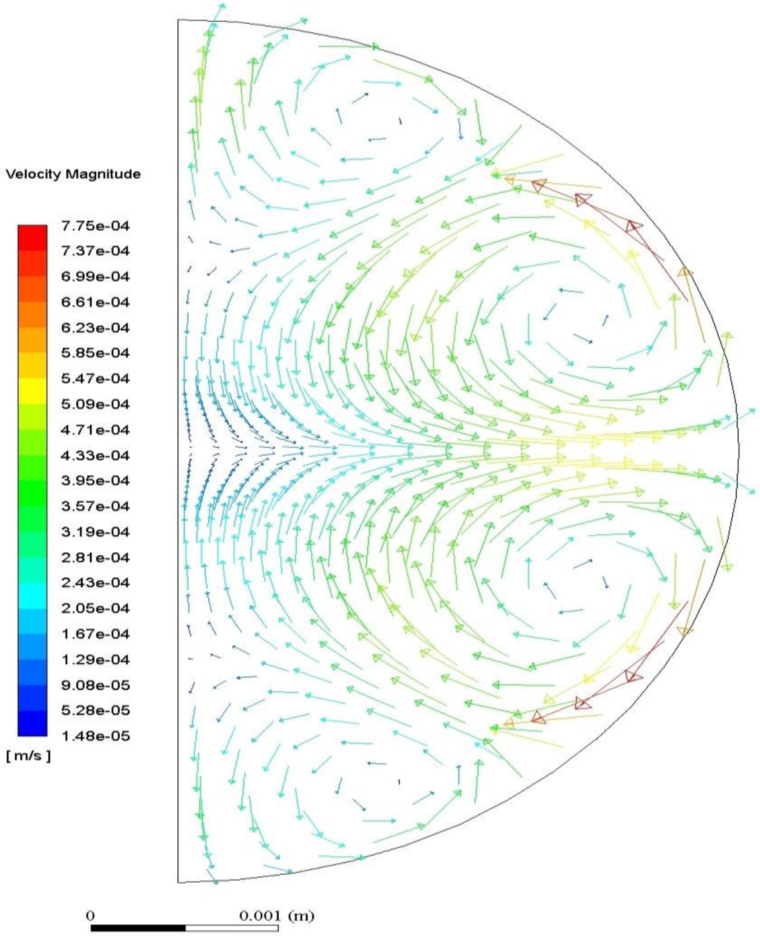

Supplementary Figure 2. The fluid flow velocity distribution in a 6.0 mm diameter $Ti_{39.5}Zr_{39.5}Ni_{21}$ liquid at 990 K when the sample was cooled with a 5.7 V positioner and heater off condition. As is apparent from the color codes and arrow sizes, the flow velocity is maximum along the surface of the sample in a direction about 40^0 from the equator.

Supplementary References

37. Hyers R. W., Matson D. M., Kelton K. F., and Rogers J. R. "Convection in containerless processing". *Ann. N. Y. Acad. Sci.* **1027**, 474 (2004).

38. Bracker G. P., Baker E. B., Nawer J., Sellers M. E. Gangopadhyay A. K., Kelton K. F. Xiao X., Lee J., Reinartz M., Burggraff S., Herlach D. M., Rettenmayr M., Matson D., Hyers R. W. "The effect of flow regimes on the surface oscillations during electromagnetic levitation experiments". *High Press. High Temp.* **49**, 49 (2020).
